# Supplementary material for: Synaptic and circuit mechanisms prevent detrimentally precise correlation in the developing mammalian visual system
Source: eLife. 2023 May 22;12:e84333. doi: 10.7554/eLife.84333 (PMC10202458; doi:10.7554/eLife.84333)
Supplement: Figure 1—figure supplement 1—source data 1. [file elife-84333-fig1-figsupp1-data1.zip › DevelopmentOfThalamocorticalNeurons/README.html]

# Database of conductance-based models of thalamocortical neurons

The current database contains only models of thalamocortical relay
neurons (TC) in the mouse visual thalamus (dorsal lateral geniculate
nucleus) recorded at postnatal day 7 (P7).

### Software

The model of TC neuron is implemented in `NEURON`
simulator.

```
pip3 install --user neuron
```

### Preparations

All necessary `NEURON` modules are located in
`mods` directory and borrowed from Iavarone
et al. (2019). You must compile them before any simulation.

```
nrnivmodl mods
```

### The basic model

Even adult TC neurons are relatively electrically compact (Sherman
and Guillery, 2004; Bloomfield and Sherman, 1989). Developing TC neurons
have shorter and thicker processes (Charalambakis et al., 2019; El-Danaf
et al., 2015). That allows the usage of a conductance-based
”pen-and-ball” two-compartment model with a single segment for the
somatodendritic compartment and a multisegment compartment for an axon.
The model is implemented as a single class in `cell.py`.

### The database

The current database `P07-selected-checked-gmin.json`
(last updated 2022-09-21) contains fully validated and human-evaluated
286 sets of model parameters.

### Try a neuron model in the database

To try neurons in the database in the standard current-clamp
protocol, you can run `cell.py` with at least two command
line arguments: name of the database file and neuron ID. The third
optional command line argument will be interpreted as the amplitude
value of the current-clamp protocol. **Note**
`cell.py` does not read initial conditions for steady-state
from the database, and therefore transient dynamics are expected.

Examples

| `python cell.py P07-selected-checked-gmin.json 2 0.1` | `python cell.py P07-selected-checked-gmin.json 280 0.1` |
| --- | --- |
|  |  |

### Citation

Please use DOI:
10.5281/zenodo.7312024 as the reference for the database.
