# Supplementary figures and images for: Synaptic and circuit mechanisms prevent detrimentally precise correlation in the developing mammalian visual system
Source: eLife. 2023 May 22;12:e84333. doi: 10.7554/eLife.84333 (PMC10202458; doi:10.7554/eLife.84333)

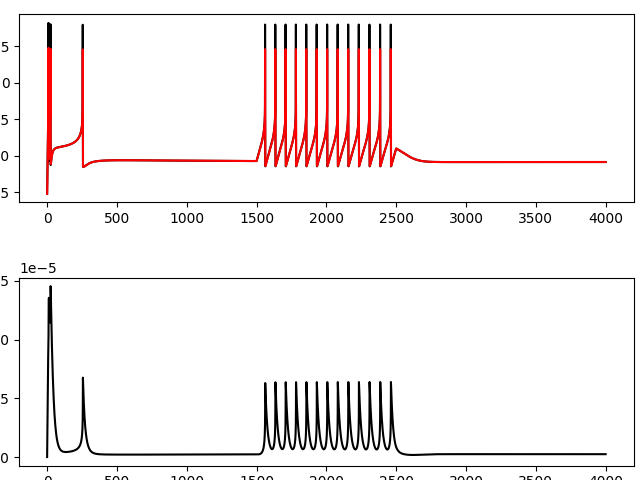

Supplement: Figure 1—figure supplement 1—source data 1. [file elife-84333-fig1-figsupp1-data1.zip › DevelopmentOfThalamocorticalNeurons/examples/Figure_1.png]

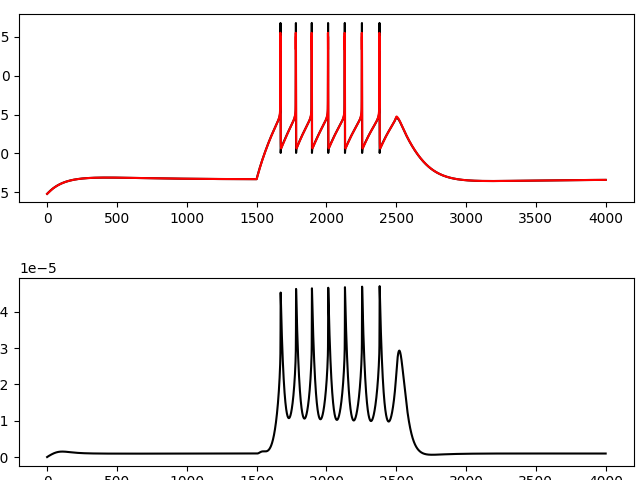

Supplement: Figure 1—figure supplement 1—source data 1. [file elife-84333-fig1-figsupp1-data1.zip › DevelopmentOfThalamocorticalNeurons/examples/Figure_2.png]
